# Piloting the Inclusion of the Key Populations Unique Identifier Code in the South African Routine Health Information Management System: Protocol for a Multiphased Study

**DOI:** 10.2196/55092

**Published:** 2024-09-06

**Authors:** Mashudu Rampilo, Edith Phalane, Refilwe Nancy Phaswana-Mafuya

**Affiliations:** 1 South African Medical Research Council/University of Johannesburg (SAMRC/UJ) Pan African Centre for Epidemics Research (PACER) Extramural Unit Johannesburg South Africa; 2 Department of Environmental Health Faculty of Health Sciences University of Johannesburg Johannesburg South Africa

**Keywords:** key populations, unique identifier code, biometric, biometrics, alphanumeric code, routine health management information system, health management, management, protocol, mixed methods study, South Africa, human immunodeficiency, immunodeficiency, HIV, AIDS, transgender, epidemiology, data extraction, HIV transmission

## Abstract

**Background:**

The global community has set an ambitious goal to end HIV/AIDS as a public health threat by 2030. Significant progress has been achieved in pursuing these objectives; however, concerns remain regarding the lack of disaggregated routine data for key populations (KPs) for a targeted HIV response. KPs include female sex workers, transgender populations, gay men and other men who have sex with men, people who are incarcerated, and people who use drugs. From an epidemiological perspective, KPs play a fundamental role in shaping the dynamics of HIV transmission due to specific behaviors. In South Africa, routine health information management systems (RHIMS) do not include a unique identifier code (UIC) for KPs. The purpose of this protocol is to develop the framework for improved HIV monitoring and programming through piloting the inclusion of KPs UIC in the South African RHIMS.

**Objective:**

This paper aims to describe the protocol for a multiphased study to pilot the inclusion of KPs UIC in RHIMS.

**Methods:**

We will conduct a multiphased study to pilot the framework for the inclusion of KPs UIC in the RHIMS. The study has attained the University of Johannesburg Research Ethics Committee approval (REC-2518-2023). This study has four objectives, including a systematic review, according to the PRISMA (Preferred Reporting Items for Systematic Reviews and Meta-Analyses) guidelines (objective 1). Second, policy document review and in-depth stakeholder interviews using semistructured questionnaires (objective 2). Third, exploratory data analysis of deidentified HIV data sets (objective 3), and finally, piloting the framework to assess the feasibility of incorporating KPs UIC in RHIMS using findings from objectives 1, 2, and 3 (objective 4). Qualitative and quantitative data will be analyzed using ATLAS.ti (version 6; ATLAS.ti Scientific Software Development GmbH) and Python (version 3.8; Python Software Foundation) programming language, respectively.

**Results:**

The results will encompass a systematic review of literature, qualitative interviews, and document reviews, along with exploratory analysis of deidentified routine program data and findings from the pilot study. The systematic review has been registered in PROSPERO (International Prospective Register of Systematic Reviews; CRD42023440656). Data collection is planned to commence in September 2024 and expected results for all objectives will be published by December 2025.

**Conclusions:**

The study will produce a framework to be recommended for the inclusion of the KP UIC national rollout. The study results will contribute to the knowledge base around the inclusion of KPs UIC in RHIMS data.

**Trial Registration:**

PROSPERO CRD42023440656; https://tinyurl.com/msnppany

**International Registered Report Identifier (IRRID):**

PRR1-10.2196/55092

## Introduction

### Background

HIV/AIDS continues to be a leading global health tragedy regardless of international and local initiatives to address the epidemic [1]. Globally, it is estimated that 38.4 million people are living with HIV at the end of 2021 [2]. According to the sixth South African National HIV Prevalence, Incidence, and Behavior survey, South Africa had approximately 7.8 million people living with HIV, indicating 12.7% HIV prevalence in 2022 [3]. Globally, key populations (KPs) and their sexual partners accounted for 70% of HIV infections in 2021 [4]. In South Africa, HIV prevalence among KPs varies noticeably, ranging from 60% among female sex workers, 37.5%-43% in men who have sex with men and transgender people, to 21.8% in people who inject drugs [5-9].

South Africa is among the countries that have committed to the Global AIDS Strategy 2021-2026 to end AIDS as a public health threat by 2030 [10]. Monitoring progress toward the 2030 agenda for sustainable development, endorsed in 2015, faces challenges regarding data disaggregation [11]. To reach these targets, a vigorous routine health information management system (RHIMS) must provide disaggregated data for proper planning, resource allocation, progress reporting, and accountability [11,12]. In South Africa, RHIMS does not include a unique identifier code (UIC) for KPs. Therefore, routine data cannot be disaggregated by the latter. There is thus a necessity for improving the current RHIMS in South Africa to guarantee KP data disaggregation to improve targeted resource allocation reporting and accountability inclusive of KPs. To address this deficit globally, in 2017, the World Health Organization (WHO) launched consolidated guidelines on the adoption and implementation of UIC for improved person-centered HIV patient monitoring and case surveillance [13-15]. However, less than one-third of Sub-Saharan Africa (SSA) countries, including Kenya, Uganda, Ghana, Mali, Burkina Faso, Togo, Burundi, Liberia, and Malawi, have adopted those guidelines and started implementing KPs UIC through alphanumeric codes and biometric systems [16-21].

Reports from those countries cited issues, such as knowledge of the technology behind biometric devices and their benefits, trust in health care providers, data privacy, and confirmed confidentiality, as critical facilitators to biometric UIC uptake among KPs [22-26]. Barriers noted included generalized fear of the unknown, personal data might end up in the hands of police, doubt about the technology implications, interrupted power supply, poor network coverage, system technical glitches, such as system failures, computer or software errors, lack of technical expertise, shortage of resources, poor funding, and maintenance cost of UIC systems [27-34]. Some studies suggested combining biometric fingerprint identification with alphanumeric codes to enhance feasibility, particularly in African rural areas. However, alphanumeric codes may lead to duplicates as some clients may change addresses, phone numbers, and names or may provide different spellings, or incorrect date of birth different from what they used during admission.

South Africa has a strong economy, advanced information and communication technology infrastructure, and reasonable advocacy for KP’s rights compared to some countries that have adopted the KP UIC. The adoption of KPs UIC in South Africa must be acceptable, affordable, and feasible, given the well-resourced status. This protocol pursues to undertake a comprehensive assessment of the prevailing status of RHIMS in SSA, appraising the level to which the incorporation of UICs for KPs has been embraced, as well as an assessment of how the current RHIMS is designed for capturing and monitoring HIV in South Africa and what evidence can be generated from it to guide the incorporation of KPs UIC into RHIMS.

### Research Questions

The following research questions will be addressed:

- What is the status quo on existing RHIMS that have incorporated KPs UIC in SSA?
- Does the monitoring and evaluation documents, tools, and guidelines incorporate UIC for KPs in South Africa?
- How is the current RHIMS designed for capturing and monitoring HIV in South Africa?
- How can the KP UIC be incorporated into RHIMS?

## Methods

### Overview

This is a multiphased study including a systematic literature review (objective 1), policy document review and in-depth interviews with stakeholders (objective 2), exploratory data analysis for the deidentified HIV data set (objective 3), and finally, the piloting of the RHIMS framework to understand how the KPs UIC can be incorporated in RHIMS (objective 4).

The study is part of a doctoral study by the first author which has attained the University of Johannesburg (UJ) Higher Degrees Committee and UJ Research Ethics Committee (REC) approval (REC-2518-2023). The doctoral study is linked to 2 larger studies in the South African Medical Research Council/UJ Pan African Centre for Epidemic Research Extramural unit, namely harnessing big heterogeneous data to evaluate the potential impact of HIV responses among KPs in generalized epidemic settings in SSA (REC-1504-2022) and assessing COVID-19 impacts on HIV prevention and treatment continuum (REC-1781-2022), which also obtained ethics approval from UJ REC.

### Methods for Objective 1

#### Aim

To conduct a systematic review of existing gray and published literature on the RHIMS and how the data is captured in SSA.

The methods for this objective consist of study setting, design, study selection and screening, assessment of study quality, data extraction, and analysis.

#### Study Setting

This objective focuses on reviewing published and gray literature from January 2013 onwards, on successes, barriers, and facilitators for implementing KPs UIC in SSA.

#### Study Design

The systematic review will be conducted in line with PRISMA (Preferred Reporting Items for Systematic Reviews and Meta-Analyses) guidelines [35] and is registered with the International Prospective Register of Systematic Reviews (PROSPERO) register (registration CRD42023440656).

#### Search Strategy

Two reviewers (MR and EP) will develop a search strategy using key terms in the research questions and consultation with the UJ librarian. For each key term, a medical subject heading term will be developed and combined with Boolean operators: “AND,” “OR,” and “NOT” during the title search [36]. The medical subject headings terms and keywords will be used to develop the search strategy. The population, intervention, comparison, and outcome framework will guide the development of search terms and keywords. Electronic search databases will comprise, but not limited to Google Scholar, MEDLINE (via Ovid or PubMed), PLoS, and Medicine Web of Science. Keywords, such as “key populations,” “men who have sex with other men,” “MSM,” “female sex workers,” “FSW,” “SW,” “people who use drugs,” “PWUD,” “people who inject drugs,” ‘PWID,” “lesbian,” “gay,” “bisexual,” “transgender and queer or questioning,” “LGBTQ,” ‘district health information system,” “RHIMS,” “UIC,” “unique patient identifier code,” “biometric scanner,” “alphanumeric code,” and “SSA,” will be used to build search strategy (Multimedia Appendix 1). Gray literature will be searched from Google, Google Scholar, WHO, Global Fund, PEPFAR/USAID, government department websites, conference proceedings, policy documents, and institutional repositories of KP organizations. Additionally, we will search the reference lists of all identified reports and papers for additional studies. Two reviewers (MR and EP) will vote independently on each reference. In case of differences, a third member (RNP-M) will be asked to give input and make the final decision based on the reviews of the 2 members.

#### Quality Assessment

The critical appraisal skills program systematic review checklist will be used after the screening to assess the quality and bias in each of the selected papers. The checklist investigates the validity, precision, and generalizability of the research [37]. Using appropriate quality assessment techniques, each paper that will be included in this review will have its methodological and research quality evaluated. Two team members (MR and EP) will independently evaluate the complete text and abstracts of the qualifying papers using the inclusion and exclusion criteria, as well as a predetermined and agreed-upon score criterion for each evaluation using Covidence (Veritas Health Innovation).

#### Study Selection

Inclusion and exclusion criteria will be used to ensure the consistent exclusion of studies that do not address the research questions. The eligibility criteria will ensure that the selected and included studies have the relevant data required to answer the research questions. EndNote 21 (Clarivate Analytics) reference manager software will be used to manage the references. Covidence software for systematic reviews will be used with two reviewers voting independently on each reference. In case of differences, a third member will be asked to give input and make the final decision based on the reviews of the two members.

#### Data Extraction

A standard extraction tool generated through Covidence software will be used to capture key indicators of interest to organize eligible papers. The information to be extracted for each paper will include the author or authors, publication year, settings, country, study method, sample size, indicators of interest, population details, and data sources.

#### Data Analysis

Thematic analysis will be used to assess results from the systematic review. A narrative summary of the results will be developed, and tables will be used to show specific details of the analysis.

### Methods for Objective 2

#### Aim

To conduct a formative assessment of the RHIMS and its stakeholders with respect to the inclusion of KP UIC in terms of operations, challenges, vulnerabilities, considerations, opportunities, and policies for improvement and its policy in South Africa.

This objective will use in-depth interviews and document reviews.

#### Methods for In-Depth Interviews With Stakeholders

##### Study Setting

The stakeholder interviews will be conducted in any province in South Africa depending on where the identified stakeholder will prefer to be interviewed. Only the interviewer, interviewee, and a trained study assistant or note-taker will be present during all face-to-face interview sessions. For privacy purposes, separate and private rooms within the office premises will be used for the interview.

##### Study Design

An exploratory study design involving in-depth interviews with stakeholders will be used.

##### Study Population

The stakeholders will comprise of policy makers and managers from various institutions working closely with the KP program including the KP community, government officials, the research community, health information or data managers, health care providers, civil society organizations, private sector, nongovernment organizations, international and local funders, and political leaders.

##### Study Sample

A purposive sample size of 20 key stakeholders who work or interact with the RHIMS and KPs will be invited to take part in the interviews using a semistructured questionnaire. However, the sample size may change depending on when saturation is reached. Study participants will be aged 18 years and older; working with the RHIMS and KPs programs in South Africa.

##### Data Collection

In-depth interviews will be conducted with stakeholders using a semistructured questionnaire. A trained study assistant will assist the researcher with data collection during the interview. The interviews will be audio-recorded with the participant’s consent. Participants will be informed that participation in the study is completely voluntary, and they have the freedom to withdraw from the study at any time. Interview notes will be taken during the interviews to back up the audio-recorded data in case of equipment failure. Each interview is expected to last for 20-30 minutes.

##### Data Analysis

The qualitative data from the in-depth or semistructured interviews will be captured and analyzed using ATLAS.ti (version 6; ATLAS.ti Scientific Software Development GmbH) [38]. Data will be analyzed by content through a thematic approach. The 6 stages of the thematic approach comprising data familiarization, coding, identification, review, naming the major themes, and compiling the final report will be applied [39]. Interview transcription, translation, and coding will form part of an iterative process. For content analysis, a “directed content analysis” methodology will be followed, in which codes are chosen both prior to and after analysis [40,41]. A priori codes will be developed using ATLAS.ti software based on previous research findings and theory [38]. Coding will be done independently by 2 researchers (EP and MR). Intracoder reliability will be assessed prior to entry into the final codebook. Close-ended questions will be analyzed descriptively, reporting proportions and percentages where possible.

#### Methods for Program Document Review

Documents to be searched and analyzed will include RHIMS policies, protocols or guidelines, reports, program logs, performance ratings, funding proposals, procedures, meeting notes or minutes, and strategic reports on HIV-related data for KPs. These documents may be in the form of hard or soft (electronic) copies.

#### Inclusion and Exclusion of the Documents

##### Overview

Documents that were published in the last 10 years to date; reporting on the South African RHIMS, written in English will be included. This will include documents in both public and private domains. Documents that were published before the year 2013 and non-English will be excluded.

##### Data Collection

Documents will be searched from South African government and nongovernment organization websites. Furthermore, websites of key multilateral organizations of interest (eg, Global Fund, UNAIDS, WHO, Centre for Disease Control and Prevention Africa, and PEPFAR) will be conducted to find more information. To complement the web-based document search, relevant individuals in government and nongovernmental organizations will be contacted to request unpublished reports that are not available in the public domain. An Excel (Microsoft Corp) data extraction tool for document review activity will be used to document outcomes. The Excel spreadsheet will have column headings containing the document name, type, source of the document, the year it was released, purpose, target audience, and summary of key content related to this study.

##### Data Analysis

Qualitative document analysis will be conducted to provide insight into how various official documents recognize KPs HIV data collection and reporting. Thematic content analysis will be used to identify themes emanating from the documents that will be reviewed.

### Methods for Objective 3

To conduct an exploratory analysis of the deidentified routine program data to understand the nature and structure of RHIMS data, outputs, and the implications thereof.

#### Study Design

Using a retrospective observational descriptive design, the analysis will focus on the District Health Information System HIV program data set covering the years 2021 to 2023, aiming to summarize and interpret the data to uncover patterns, trends, and insights. The data will be sourced from the National Department of Health (NDoH) and will encompass demographic information (age, gender, geographic location), clinical data (HIV diagnosis dates, cluster of differentiation 4 counts, viral load measurements), and treatment details (antiretroviral therapy [ART] initiation dates, regimen types, adherence measures, and virological outcomes).

#### Study Population

The study population will consist of individuals whose information is captured within the deidentified HIV routine program data set sourced from the NDoH. This data set includes individuals diagnosed with HIV, with recorded demographic, clinical, and treatment details. Encompassing a diverse array of individuals across various age groups, genders, geographic locations, and clinical profiles, the population offers a comprehensive representation of how HIV data is documented within the national health management information system.

#### Sampling

Random sampling will be used to select a representative subset of records from the deidentified HIV routine program data sourced from the NDoH. The sample will include facilities from all 9 provinces and 52 health districts in the country. This method aims to ensure comprehensive coverage of the population while minimizing bias and enhancing the generalizability of the study findings.

#### Data Collection

A standard data extraction tool will be used to extract deidentified data from the HIV database as an export file. Data for this objective will be collected from the deidentified HIV routine program data set maintained by the NDoH. A data sharing agreement will be submitted to the NDoH to request deidentified HIV-related data. The data sharing agreement will be accompanied by a list of HIV-related data elements and indicators that are collected by NDoH at the facility level in South Africa. These HIV-related elements and indicators are listed in the National Indicator Data Set, which is a document outlining the list of indicators and elements introduced by the NDoH that every public health facility is required to capture and report. Data cleaning procedures will be implemented to address any missing values or inconsistencies, ensuring the integrity and reliability of the data set. Strict measures will be taken to protect patient confidentiality, including the removal of personal identifiers, and access to the data will be restricted to authorized personnel only.

#### Data Analysis

This objective will use an exploratory-descriptive approach to analyze deidentified HIV routine program data. Exploratory data analysis will be conducted using Python 3.11.4 programming language. The analysis will include univariate nongraphical (central tendency, spread skewness, and kurtosis), multivariate nongraphical, univariate graphical (histograms, stem-and-leaf plots, boxplots, and quantile-normal plots), and multivariate graphical (scatterplots, run charts, multivariate charts, Bubble charts) outputs. A data deduplication process will be undertaken to eliminate multiple records that pertain to the same individual. When unique identifiers are available, we will use exact matches to ascertain whether records belong to the same person. However, in cases where unique identifiers are absent, we will use matching algorithms to calculate the probability of multiple records belonging to the same person.

### Methods for Objective 4

To pilot the inclusion of the KPs UIC in RHIMS in South Africa using findings from objectives 1, 2, and 3.

#### Study Design

Drawing insights from objectives 1, 2, and 3, an integrated framework will be developed, by adapting the PRISMA framework. This framework will undergo a pilot phase within selected health care facilities. During this pilot, primary health care registers and clinical stationery will be adjusted to include UICs for KPs. Over the course of 1 month, these refined tools will be used to gather comprehensive data on HIV testing, ART initiation, ART outcomes, viral load collection, and suppression. To evaluate the feasibility of UIC integration into data collection tools, semistructured qualitative interview questionnaires will be administered to testers or lay counselors, data captures, and clinicians.

#### Study Setting

The framework will undergo a pilot phase in Gauteng province, specifically in the Tshwane district, where both KPs are served, and high HIV burden facilities exist. The district’s notable HIV prevalence renders it an optimal setting for assessing the framework’s effectiveness. Focusing on high-burden facilities will ensure a comprehensive representation of health care settings, offering valuable insights applicable to wider contexts.

The framework will be pilot-tested among health care providers, data captures, and HIV testers or lay counselors who are 18 years and older and employed within the selected facilities. Additionally, the population will encompass individuals accessing health care services at the selected facilities.

The individuals need to be on permanent employment or contract employment beyond the duration of the piloting to avoid participants discontinuing from the study because of employment termination. By including various personnel, the study intends to capture a wide range of experiences and perspectives, facilitating a thorough evaluation of the framework’s feasibility and effectiveness in real-world health care settings.

#### Data Collection

A semistructured questionnaire will be used to collect feedback from the pilot sites. Trained researchers will conduct face-to-face interviews with HIV testers or lay counselors, data captures, and clinicians at each pilot facility. The interviews will be recorded to ensure the accurate capture of participants’ responses and insights. Conducted in an environment conducive to open and sincere dialogue, participants will have the opportunity to provide additional comments or insights beyond the structured questionnaire. This approach allows for a comprehensive understanding of the pilot implementation process, enriching the evaluation with nuanced perspectives.

#### Data Analysis

Qualitative data analysis will be carried out following similar steps outlined in objective 2 for semistructured interviews with stakeholders.

### Ethical Considerations

Ethical guidelines will be adhered to throughout the research process. Ethics approval has been secured from the UJ for the Research Ethics Committee (UJ REC) for this study (REC-2518-2023). Furthermore, since human participants will be used in the study, several principles will be adhered to, enhancing the norms and standards of ethics, as well as aligning with the guidelines of the Protection of Personal Information Act 4 of 2013 (POPIA). These principles are elaborated further in this section. With regards to objectives 2 and 4 (stakeholder interviews and pilot site), the participants will be informed about the study’s methodology and procedures using the study information letter in line with the POPIA. The participants will further be provided with a personal information letter detailing the personal information that will be collected during the study according to the POPIA and thereafter be asked to sign the personal information consent form acknowledging that they have understood the information contained in the personal information letter. Before taking part in the study, all participants will be asked to give written informed consent. The interviews will be audio-recorded with the participant’s consent. Participants will be told that participation in the study is completely voluntary and will be allowed to withdraw their consent at any stage of the study. For privacy purposes, separate and private rooms within the office premises will be used for the interview. Only the interviewer, interviewee, and note-taker will be present during all face-to-face interview sessions. The interview will be solely done in the interest of the research topics. The interviews will commence with a brief introduction of the study objectives, introducing the interviewer, and reading the privacy declaration form. In line with the POPIA, information will not be analyzed using participant’s names, but pseudo names will be used in the analysis where verbatim quotations are applied. Since anonymized data cannot be considered completely anonymous, necessary provisions will be taken to remove any identifying information or avoid reidentification. For example, a pseudonym will be assigned to each participant, ensuring that the interview transcript does not contain any identifying information. This will also be consistent with UJ’s data sharing and processing policies for the use of secondary data.

## Results

As of June 2023, the systematic review protocol was registered with PROSPERO (CRD42023440656). The results will encompass a systematic review of literature, qualitative interviews, and document reviews, along with exploratory analysis of deidentified routine program data and findings from the pilot study. Prior to commencing the study, formal authorization will be secured to conduct stakeholder interviews and access deidentified HIV programmatic data. Data collection will commence in September 2024 and expected results for all objectives will be published by December 2025.

## Discussion

### Principal Findings

This study will significantly contribute to enhancing HIV management and addressing the epidemic among KPs in South Africa and beyond, ultimately improving health outcomes and reducing disparities. Integrating UICs for KPs into RHIMS in South Africa will enhance HIV monitoring and programming by enabling the identification of HIV trends among specific KP groups, offering insights crucial for tailoring interventions to address their unique needs. Consolidating the principal findings of all 4 objectives, this study will provide a comprehensive understanding of the UIC implementation landscape, encompassing successes, challenges, and opportunities across SSA. Furthermore, it will furnish valuable insights into the operational, technical, and policy aspects of UIC integration into RHIMS. The systematic literature review will not only shed light on RHIMS implementation challenges and facilitators for UIC integration but also garner best practices, and comparisons to prior studies, potentially expediting the implementation process while mitigating errors through lessons learned from countries that failed to incorporate UICs successfully. Formative assessments of RHIMS and stakeholder perspectives will refine strategies for engagement and policy development, fostering collaboration among diverse stakeholders and ensuring a unified approach to UIC integration. The exploratory analysis of routine program data will yield crucial insights into existing RHIMS data structures and trends, guiding further enhancements and optimizing data use for evidence-based decision-making. The integration of the KPs UIC allowing for the disaggregation of data to include marginalized KPs will contribute to the Government’s achievement of the Sustainable Development Goals 2030 agenda of leaving no one behind.

This study’s strengths lie in its comprehensive approach, involving diverse stakeholders across multiple objectives, potentially yielding valuable insights into UIC integration. However, limitations include context-specific findings, challenges related to data quality, potential implementation barriers, and scope constraints.

The future direction of this study entails extending the integration of KPs UICs into other government information management systems in South Africa and other SSA countries. Subsequent research efforts could focus on assessing the sustained impact of this integration on HIV monitoring, programming, and health outcomes among KPs, while also exploring advancements in technology and methodologies to optimize data collection and analysis within RHIMS for more effective public health interventions.

Concerning our dissemination plan, we aim to publish study findings under each objective in peer-reviewed journals. Summary reports will be distributed to key stakeholders, policy makers, and relevant organizations to ensure broad visibility and use of the study outcomes.

### Conclusions

This study will produce a framework to recommend the national rollout of KPs UIC inclusion. The results will contribute to the knowledge base on integrating KPs UIC into RHIMS data, leading to data optimization, complete reporting, and improved programming.

## Appendix

Multimedia Appendix 1 PubMed search strategy.

## Abbreviations

ART: antiretroviral therapy
KP: key population
NDoH: National Department of Health
POPIA: Protection of Personal Information Act
PRISMA: Preferred Reporting Items for Systematic Reviews and Meta-Analyses
PROSPERO: International Prospective Register of Systematic Reviews
REC: Research Ethics Committee
RHIMS: routine health information management system
SSA: Sub-Saharan Africa
UIC: unique identifier code
UJ: University of Johannesburg
WHO: World Health Organization

## Data Availability

Data sharing is not applicable to this article as no data sets were generated or analyzed during this study.
