# Supplementary material for: Piloting the Inclusion of the Key Populations Unique Identifier Code in the South African Routine Health Information Management System: Protocol for a Multiphased Study
Source: JMIR Res Protoc. 2024 Sep 6;13:e55092. doi: 10.2196/55092 (PMC11415720; doi:10.2196/55092)
Supplement: Multimedia Appendix 1 [file resprot_v13i1e55092_app1.pdf]

Pubmed

(("Transgender"[tiab] OR "Transgender population*"[tiab] OR "Transgender people"[tiab] OR "Transgender person*"[tiab] OR "TG"[tiab] OR "LGBTQ"[tiab] OR "Gay"[tiab] OR "Lesbian*"OR "Men who have sex with men"[tiab] OR "MSM"[tiab] OR "Sex work*"[tiab] OR "SW"[tiab] OR "people who use drugs"[tiab] OR "people who inject drugs"[tiab] OR "PWUD"[tiab] OR "PWID"[tiab] OR "vulnerable population*"[tiab] OR "key population*"[tiab] OR "hidden population*"[tiab] OR "vulnerable people*"[tiab] OR "Priority population*") AND ("unique"[tiab] OR "personal"[tiab] OR "identifier"[tiab] OR "code*"[tiab] OR "biometric*"[tiab] OR "UIC"[tiab] OR "UPI"[tiab] OR "Unique identifier code"[tiab] OR "Inclusion"[tiab] OR "Unique personal identifier"[tiab] OR "Finger print"[tiab] OR "health information management system"[tiab] OR "health management information system"[tiab] OR "routine health management information system"[tiab] OR "health information system"[tiab] OR "District Health Information System"[tiab] OR "DHIS"[tiab] OR "DHIS2"[tiab] OR "RHIMS"[tiab] OR "RHMIS"[tiab] OR "Electronic register"[tiab] OR "Electronic information system"[tiab] OR "HPRS"[tiab] OR "Electronic medical record"[tiab] OR "EMR"[tiab] OR "Surveillance"[tiab] OR "cascade")) AND ("sub Saharan Africa"[tiab] OR "Kenya"[tiab] OR "Ghana"[tiab] OR "Zimbabwe"[tiab] OR "Malawi"[tiab] OR "Lesotho"[tiab] OR "Mozambique"[tiab] OR "Nigeria"[tiab] OR "Angola"[tiab] OR "Namibia"[tiab] OR "eSwatini"[tiab] OR "Ethiopia"[tiab] OR "Botswana"[tiab] OR "DRC"[tiab] OR "Zambia"[tiab] OR "Mali"[tiab] OR "Burundi"[tiab] OR "Cameron") AND ((y_10[Filter]) AND (humans[Filter]) AND (english[Filter]))
